# Coronavirus Trauma and African Americans’ Mental Health: Seizing Opportunities for Transformational Change

**DOI:** 10.3390/ijerph18073568

**Published:** 2021-03-30

**Authors:** Lonnie R. Snowden, Jonathan M. Snowden

**Affiliations:** 1 School of Public Health, University of California, Berkeley, CA 94610, USA; 2 Department of Obsterics & Gynecology, Oregon Health & Science University and Portland State University, Portland, OR 97201, USA; snowden@ohsu.edu

**Keywords:** COVID-19, African Americans, mental health, disaster response, mental health services, mental health policy

## Abstract

The COVID-19 pandemic is a natural disaster of historic proportions with widespread and profound psychological sequelae. African Americans fall ill and die more than whites from COVID and more survivors and loved ones face psychological risk. African Americans also experience greater personal, social, and financial stress even when not personally touched by COVID illness, and they are again vulnerable as COVID diminishes African American community’s capacity for mutual support. Enactment of the American Rescue Act of 2021 can moderate if not eliminate African Americans’ greater adversity and greater psychological challenge; other provisions can move the mental health treatment system beyond its previous failure to reach African Americans as it constructively responds to the crisis that COVID presents. From outreach through trusted community actors and institutions for meeting African Americans’ needs of varying intensity and duration, and by providing a spectrum of evidence supported interventions—culturally adapted as needed—newfound success can mark a turning point toward new approaches and lasting success.

## 1. Introduction 

The coronavirus pandemic is natural disaster so great that historians judge it among humanity’s leading mass traumas over the past hundred years. Widespread illness has spread fear of falling ill and dying, anguish over the illness and loss of loved ones, disruption in social and economic institutions and personal day-to-day personal routines, increased social isolation and economic insecurity, and short-run uncertainty and unsettled long term prospects. The virus’s impact has been uneven and African Americans have suffered disproportionately

Trailing disasters are wrenching secondary effects including trauma, psychological distress, and mental illness [1,2]. Expressions of trauma can be dramatic. As one of several bubonic plague pandemics swept Europe unchecked in the 14th century (the “Black Death”), survivors guilt prompted mass displays of self-flagellation and panic and drove frenzied flight from affected areas. Scapegoating triggered violence against low-ranking people and groups held responsible for otherwise inexplicable mass suffering [3]. Quoting Petrarch Christiakas [4] depicts widespread despair: “Our former hopes are buried with our friends”. “Last losses are beyond recovery and death’s wound beyond cure”. Modern public health and medicine provide reassuring science-based knowledge but pervasive psychological destabilization persists, as does pandemic’s greater impact on marginalized groups [5]. 

Social and political forces continue to shape structural inequities which channel infectious diseases’ impact [3], including psychological distress, more to marginalized groups. African Americans have suffered especially from COVID [6,7] and African Americans also have suffered more from trauma, psychological distress and mental illness resulting from COVID, even as these outcomes burden African Americans with more health and greater functional disability [8]. As illness places more African Americans at risk for psychological distress, so do higher death rates burden more African Americans with grief. Disproportionate social and financial vulnerability increases African Americans’ levels of stress while social bonds are strained by more caregiving needs, even as distancing requirements further undermine African American community’s capacity for increasingly needed mutual support. 

The mental health treatment system is challenged to provide assistance but in the past, African Americans have failed to receive mental health treatment in numbers commensurate with African Americans rates of mental illness [8]. Historically rooted, systemic inadequacies persist as the system now confronts African Americans’ burgeoning and diverse mental health-related needs. The system must restructure to better reach out to African Americans and provide newly required, culturally appropriate mental health assistance to an already underserved African American population. If it can redress historical inadequacies as the then Institute of Medicine foresaw: “the disaster recovery process offers a series of unique and valuable opportunities to improve on the status quo” [9]. Failure to overcome historical inadequacies will perpetuate or worsen the consequences of previous neglect [6].

In what follows we review literature on African Americans and COVID, trauma and mental illness and other psychological distress and outreach to African Americans for mental health treatment responsive to COVID-related and other needs. We begin by considering the scale of the COVID pandemic in a context of other disasters and considering its destabilizing social and psychological effects. We then consider how COVID sickness potentially has a greater psychological impact on African Americans. We continue by addressing COVID’s undermining African Americans resilience, bringing greater personal, social, and financial stress from COVID amid diminishing prospects for much needed mutual support. Finally, we describe key aspects of the health care system and offer proposals for constructive response to the COVID crisis—highlighting provisions of the recently enacted American Rescue Plan Act of 2021 congruent with our proposals’ aims. We suggest how, moving forward, success in bringing mental health services to African American communities can mark COVID as a turning point toward new approaches and better success. We call on evidence from representative national surveys wherever possible to supplement an evolving peer-reviewed literature which cannot always keep abreast of an unanticipated and changing national and worldwide crisis.

### The COVID Pandemic as Natural Disaster

In addition to being an infectious disease outbreak, the coronavirus pandemic is a natural disaster akin to earthquakes, hurricanes, tsunamis, or floods; like other disasters it causes secondary psychological distress in the wake of focal devastation. Like other pandemics but unlike geographically circumscribed disasters from a singular event, COVID illness is widely dispersed and continuing. Polio, the early HIV epidemic, Ebola, other coronavirus outbreaks (e.g., MERSA, SARS), and Swine Flu were more virulent but and more limited and proved less disruptive to society at large [10]. In deadliness and scope, coronavirus approximates once-in-a-generation pervasive, lethal, and disruptive episodes such as the 1918 influenza pandemic [11]. Regarding COVID’s social significance some analysists conclude: “Due to the scope of its devastation, the novel coronavirus pandemic may be considered a “transforming event” comparable to the 9/11 attack, although the damage wrought by this pandemic has been many times more severe in all respects”. [12]

Natural disaster’s secondary effects include PTSD, depression, anxiety, substance use disorders, somatic complaints and suicide [1,2,9] and, as discussed later, African Americans are at greater risk than whites [2]. Psychological adversity persists even as physical threat subsides from widespread dislocation and as new behavioral disorders arise and latent or remitted mental disorders are activated. For most affected people any psychological adversity will be transient but for many it will be long-lasting and bring about the significant functional impairment that places mental disorders among the leading causes of disease burden worldwide [13]. 

## 2. COVID Illness as a Source of Psychological Distress in The U.S. and Worldwide

COVID illness itself is a risk factor for serious psychological distress [14,15,16,17] and the risk of diagnosable secondary mental illness in the aftermath of COVID diagnosis is about 18% [18]. COVID-precipitated death elicits strong grief reactions in loved ones [19]: untimely death as from COVID was, in one study, rated as the respondent’s worst among traumatic experiences [20]. Social isolation can prolong and deepen grief by denying grieving loved ones the solace of communal grieving, a concern especially for older people already distressed from limited social contact [21]. The too-little recognized impact of grief led one research team to a grim forecast: “We predict that pandemic-related increases in pathological grief will become a worldwide public health concern” [19]. 

In the population at large the pandemic’s many and diverse psychological challenges are such that fully 78% of respondents from one study reported pandemic stress as significant [22]. Worries include becoming infected oneself or infecting other people, especially loved ones. Concern increases as employment is interrupted or lost and financial uncertainty increases [23]. Social distancing mandates and disruption of social life and daily routines [23] increase risk of loneliness and social isolation, major risk factors for adverse mental and physical health outcomes, especially among older adults [24,25]. Established structures for day-day day living are interrupted and their return is postponed indefinitely—sometimes with no prospect of return. Disruption is intermittent as new infection comes in waves and restrictions are intermittently applied and eased [14]. In response, over half of adults reported problems with sleeping or eating, increased alcohol use, or worsening chronic health conditions due to worry or stress from COVID [22]. Self-reported mental health suffers: 53% of adults reported COVID adversely effected their mental health in July 2020, an increase from 32% in March [23].

Psychological distress secondary to COVID is problematic worldwide. The UK’s population prevalence of clinically significant psychological distress rose from 19% before the pandemic to 27% in April 2020 [26], and comparable levels have been found in other countries [27]. One analysis of U.S. Centers for Disease Control (CDC) surveys revealed an even greater rise in anxiety and depression disorder symptoms: they increased from 11.0% in early 2019 to 41.1% in January of 2021. 

## 3. African Americans: More COVID Illness Yields Greater Psychological Distress

Racial disparity in COVID extends across the progression of the infection from acquisition to progression to mortality, and each stage brings for African Americans greater secondary psychological risk. Greater risk begins with African Americans’ three times greater than whites liklihood to become ill from COVID [28,29] which itself promotes distress and mental illness [18].

African Americans’ infections are more severe: African Americans are about 4.6 times more likely to be hospitalized than whites [28,29]. Intensive care confinement and mechanical assistance for breathing, when required, can traumatize patients, and survivors are more likely to develop enduring depression and anxiety [30,31]. Higher hospitalization rates indicate that African Americans are disproportionately exposed to hospital-based traumatic experience.

Risk of psychological adversity is greater still because COVID-19 symptoms can persist for months, perhaps continuing indefinitely, among “COVID long haulers” [30,32]. Scar tissue on the lungs can cause long-term breathing problems, and heart muscle damage can cause weaknesses even in mildly symptomatic people. In conjunction with African Americans already higher rats of cardiovascular disease and respiratory disease—along with other co-morbidities including malignant neoplasms, cerebrovascular disease, diabetes, and obesity [33]—experiencing longer term and more severe COVID can further increase African Americans’ prospects for serious psychological distress. 

African Americans higher COVID mortality rates [28] assure that more loved ones are left in grief, exposing more African Americans to COVID severe grief reactions [19]. Their grieving is fraught because, as discussed below, it is accompanied by disproportionately many economic, health-related and other stressors which further increase African American’s potentially adverse psychological secondary COVID response [34].

Decisively suppressing COVID infection and sequelae will leave behind enduring harm but will reduce new African Americans’ COVID suffering and the psychological distress it activates. Immunization is essential but widespread acceptance must surmount a barrier: African Americans’ vaccine hesitancy, rooted in historical and contemporary experiences of racism that fuel mistrust of health systems and interventions [35]. The recently enacted American Rescue Plan Act of 2021 [36] funds vaccine confidence-building, information, and education activities and these should include messaging tailored to African American communities. Trusted leaders who respectfully convey accurate information [35] can address legitimate sources of vaccine hesitancy. Vaccinating African Americans to reduce COVID infection rates is essential to reducing their COVID-related psychological distress.

## 4. African Americans Challenged Resilience: Greater Stress Amid an Overloading of Resources for Mutual Support

African American individuals and communities have been injured especially economically and socially by COVID, which deprives African Americans of crucial requirements for a psychologically resilient disaster response. Playing on structural inequalities, COVID limits African American’s access to: “key predictors of the development and course of disaster-related mental illness: post-disaster life stressors and social support” [2], leaving African Americans disproportionately vulnerable to COVID’s secondary psychological impact. 

*African Americans’ Greater COVID-Related Stress.* Economic decline has hit African Americans especially hard because African American workers are overrepresented in occupations that are most affected by the COVID-19 pandemic—food services, customer service, and sales and office support [28] such that African American unemployment has risen disproportionately [28]. African American business owners and their employees too have suffered greatly: 41% of Black-owned businesses closed, a rate 2.5 times greater than white owned businesses [37,38].

Fifty-two percent of African American workers report that the coronavirus outbreak is major threat to their financial situation compared with 43% of whites [39]. To meet financial responsibilities despite lost income key resources are personal and family wealth: owning a home, having savings and investments, or having other and other financial assets for borrowing. Yet, white families possess 6.7 times more wealth than African American families [40]. Due to COVID, African Americans are about 1.7 times as likely as whites to say they had trouble covering usual household expenses and 2.5 times as likely to report not having enough to eat, 3 times as likely to say they were not caught up on rent [41]. 

Socioeconomic disadvantage also confers non-economic COVID-related difficulties and concerns. Thirty-nine percent of jobs held by African Americans are in occupations where workers are at risk. From having more sick and vulnerable co-workers as well as more family, friends and neighbors [42,43] African Americans are twice as likely as whites to know someone who has been hospitalized or has died because of COVID- [39], and they are about 1.4 times more likely to be very concerned about unknowingly spreading COVID to others [39]. 

African Americans’ risk of COVID-related PTSD is compounded by high rates of previous trauma [44] due to personal and family adversity. African Americans are more likely to be victims of or witness violence and to have friends or relatives who become victims of violence [45]. They also have experienced more traumatic childhood events [46], and levels of current PTSD are higher than whites [47]. Previous trauma predicts responding to a new disaster with PTSD [2]. 

Distinctive stressors arise from racial stereotypes which African Americans must navigate. African Americans perceived that other people were more likely to become uncomfortable with their race during the pandemic and were 8 times more likely than whites to report fearing that others might become suspicious if they wore a mask in public [48]. Perversely, long-standing and pervasive stereotypes of African Americans as criminal can add another layer of fear and psychological strain. 

*African Americans’ Threatened Social Cohesion and Mutual Support*. Counties with heavier African American representation have considerably more COVID cases and higher COVID death rates [37,42,43] pointing to significant social and community impact from COVID. Manifestations include burdened and disrupted social bonds and prosocial community norms from COVID’s spread, reducing family members’ and friends’ capacity for increasingly needed mutual support. 

Greater COVID risk places new demands on nurturing emotional bonds as family and friends must assist more loved ones in need. Mass incarceration means that non-incarcerated African Americans are 50% more likely to have an incarcerated family member who is at great COVID risk [49]. Children’s educational needs become paramount as schools are closed due to COVID and vulnerable seniors in multigenerational households require time and attention [28]. These and other demands accumulate and further tax African American families’ and friends’ capacity to support each other, even as African Americans must more often isolate and socially distance from family and friends due to higher COVID rates [28].

Under significant stress themselves, family and friends can unwittingly transmit pandemic-related stress [50] rather than providing emotional support. Indeed, they can directly communicate their own depression and anxiety as contagion effects ripple through social networks [51]. Stressed social bonds can fray social cohesion community wide such that African Americans risk of depression and other mental health problems increases [52]. These network- and community- level adversities, more prevalent for African Americans, reduce availability of nurturing interaction in strong and positively toned relationships community wide. Economic and social pressures appear ultimately to translate into disparities in psychological distress: CDC’s Household Pulse Surveys finds that, between August and December of 2020, African Americans reported more symptoms of anxiety or depression than whites on 20 of 21 reporting periods [53,54].

The American Rescue Plan Act of 2021 [36] makes inroads in lowering risk and enhancing mutual support: key provisions benefit African Americans disproportionately due to enduring structural inequalities. The Act provides a one-time, direct cash payment to individuals and extended Federal unemployment benefits. It expands tax credits for low income workers and for children and assists low-income households with rent, utility and nutritional assistance. The Act widens opportunities for paid sick and family leave and provides education and childcare benefits. Small business finds financial relief and agricultural relief benefits black farmers especially. These and other provisions can moderate economic stressors impinging disproportionately on African Americans and help to restore greater social cohesion and mutual support. 

Providing meaningful short term relief must be implemented equitably, and Americans African Americans experienced more benefit delays and failures than whites from the previous Coronavirus Aid, Relief, and Economic Security (CARES) Act [55]. Furthermore, even if the American Plan Rescue Act is equitably implemented, more African Americans than whites will experience greater COVID economic and social risk as effects of previous exposure endure and ongoing stress and social disruption persist. Nor can the Act’s social and economic provisions meet professional assistance needs of those who have already succumbed to new or recurrent mental illness or experience long-term psychological distress. 

## 5. Restructuring the Mental Health Treatment System to Reduce African Americans’ Barriers to Care

Mental disorders and serious psychological distress require mental health intervention to relieve suffering and permit successful community functioning. Surging use of hotlines [56] suggests sharp increases in needed assistance. Yet barriers have prevented African Americans experiencing mental illness from receiving mental health care in the past, and there is little reason to believe that longstanding barriers will fall as COVID increases levels of need. 

World Health Organization (WHO) standards for psychological and mental health support following disasters call for a comprehensive response [57]: “Conduct outreach, surveillance, screening; task sharing to expand capacity; adopt evidence based interventions”. Recommended interventions must be culturally adapted as circumstances warrant [58,59]. WHO guidelines must be applied on a scale large-enough to improve population health equity—or at least not to exacerbate ongoing inequity [60,61]. 

Financial support for existing and new mental health programming will grow as states receive increased mental health block grant awards under The American Rescue Plan Act [36] for an enhanced COVID response. When formulating a response, mental health officials should consult WHO guidelines for direction and emphasize meeting African Americans’ needs. Other provisions which are discussed below can support innovative programming initiatives.

*Implementing Crisis Response Services for African Americans.* Evidence-based crisis response services must be deployed to identify African Americans who struggle with psychological challenges brought on by COVID stressors, informing them about strategies for recognition of psychological distress and intervening for short-term symptom relief [62,63]. These include Psychological First Aid (PFA) for acute response, and Skills for Psychosocial Recovery (SPR). People in continuing distress may require Trauma-Focused Cognitive Behavior Therapy and other interventions of demonstrated benefit [62]. Persons with other full-blown mental illnesses should be identified and referred for evidence based treatment delivered by mental health specialists.

Well-suited to implementing such interventions are The American Rescue Plan Act’s [36] provisions for extending community-based mobile crisis response teams--trained in trauma-informed care and which include a behavioral healthcare professional. Mobile crisis intervention services should be deployed in African American communities and, wherever deployed, they should reach out to African American individuals to identify African Americans’ and COVID-related mental health needs.

*Mobilizing Healthcare Delivery to Detect and Treat African Americans’ COVID-Related and Other Mental Health Problems.* African Americans’ higher COVID and chronic disease prevalence rates should bring more African Americans into the healthcare system for general medical treatment where stigma interferes less with detection and treatment of mental health problems [64]. Team-based approaches for healthcare settings, which integrate behavioral health into primary care, have gained increasing acceptance for detection and treatment of mental health problems [65]. Research shows that African Americans’ mental health treatment increases in response to integrated approaches [66], allowing for subsequent improvement in mental illness and comorbid general medical conditions. Integrated care models should be adopted wherever African Americans receive healthcare for detection and management of COVID psychological distress. 

Expressly designed to provide integrated care are Certified Community Behavioral Health Clinics programs receiving $420 million in The American Rescue Plan Act [36]. A generational investment in mental health care, Community Behavioral Health Clinics emphasize care coordination, use of evidence-based practices and increasing opportunities for access to integrated health and mental health care. They can disproportionately address African Americans’ COVID mental health needs due to African Americans’ higher COVID rates and higher rates of chronic disease, and they should embrace as core organizational commitments outreach to African American individuals and communities and recognition and treatment of their mental health problems.

*Moving Mental Health Treatment Systems toward Better Engagement With African American Communities.* Outreach must increase, conducted by accepted, well connected figures in African American communities who can reach individuals and families in psychological distress. Outreach efforts should engage with community opinion leaders and trusted community institutions for consultation about COVID-related mental health concerns. Community health workers are widely employed by health plans and community clinics where, as members of comprehensive delivery teams, they act as system navigators, educators, and outreach and referral agents [67]. Supported by provisions of the American Rescue Plan Act [36] for increasing their ranks, suitably trained community health workers can identify and engage with distraught community members, providing frontline crisis assistance and referral when needed for longer-term mental health treatment. 

Not-for-profit community-based organizations—non-governmental, civil society, faith, or other grassroots organizations with a community service mission [67]—have played meaningful roles by reaching out to African American communities and reducing mental health treatment disparities [68]. Developed in consultation with community stakeholders, their strategic plans can incorporate as high priority concerns promoting COVID-related mental health awareness along with outreach and referral of persons in need. In other areas of health, African American-owned and –operated community venues have shown promise for reaching community members where they are and delivering culturally tailored health prevention messaging (e.g., through churches as barbershops) [69]. 

Thousands of nonprofit hospitals have been encouraged to become community hubs in return for state, federal, and local tax relief [70]. Not-for-profit health plans and hospitals should assess levels of COVID-related psychological distress in African American communities in their mandated community health needs assessments [71].

*Combating Stigma*: *Reframing and Integrating Mental Health Thinking Into African Americans’ Everyday Life*. Due in part to stigma [64], mental illness and mental health concepts are unappealing for many African Americans. COVID’s biomedical character and widely shared social, economic, and psychological concerns afford opportunities to offer reorientation of African Americans’ perspectives on mental health-related problems and psychological assistance. Heightened awareness of personal vulnerability presents occasions to teach and reinforce widely applicable coping skills for cushioning the impact of psychosocial adversity and managing day to day struggles more successfully. COVID can legitimize confronting personal problems and thinking strategically about to solve them in an environment of less stigma and skepticism than typically greet recommendations for improved mental health. Interventions presented as stress and coping self-improvement [72] and psychoeducation [73] can overcome stigma as it reorients mental health service delivery in a more African American- friendly manner. 

## 6. Conclusions

Transformational change is necessary and feasible in response to COVID and its psychological sequelae, a crisis which demands that the mental health system to reorient itself with better outreach and more flexible delivery to better meet African Americans increasing mental health needs. In a 2015 report on successful community rebuilding following disasters [9] the former Institute of Medicine emphasized a potential for necessary system transformation: proclaiming the short sightedness of a return to pre-event conditions and potential for understanding the disaster recovery process as a unique opportunity to improve the status quo.

Already COVID has forced policy makers to launch initiatives that were barely conceivable before COVID to meet this historic crisis. The recent, very large American Rescue Plan Act enacts measures that alleviate greater social an economic burdens borne by African Americans and that can respond to African Americans most pressing mental health needs with programming suited to their circumstances. Properly implemented and sustained, wide-ranging systems transformation in response to COVID like that called for by The World Health Organization can be realized.

## Data Availability

Not Applicable.
